# Interinstitutional collaboration for end-user bioinformatics training: Cytoscape as a case study

**DOI:** 10.5195/jmla.2017.224

**Published:** 2017-04

**Authors:** Marci D. Brandenburg, Rolando Garcia-Milian

## Abstract

**Background:**

This case study describes the value of and need for interinstitutional collaboration to train biomedical researchers in data visualization, using Cytoscape network analysis software as an example. To provide training on Cytoscape software to Yale University biomedical researchers, a collaboration was formed between the Yale Harvey Cushing/John Hay Whitney Medical Library’s (CWML’s) biomedical sciences research support librarian and the University of Michigan (U-M) Taubman Health Sciences Library’s bioinformationist, who has expertise in Cytoscape software.

**Case Presentation:**

The U-M bioinformationist offered a webinar to the Yale community, followed by a one-day onsite workshop. This collaboration allowed Yale biomedical researchers and librarians to receive training on Cytoscape software, in addition to giving the Yale librarian a collaborator for answering future questions about the software.

**Conclusions:**

This collaboration furthered the U-M bioinformationist’s role in the field as an expert in Cytoscape instruction, while also establishing the CWML as a leader in providing support for analyzing and visualizing molecular data at Yale University. The authors found this collaboration to be a successful way for librarians to fill end-user training gaps in rapidly changing fields such as bioinformatics.

## BACKGROUND

The development of high-throughput experimental technologies has given rise to the “-omics” era characterized by an exponential growth of diverse data (e.g., transcriptomics, proteomics, metabolomics). One of the most significant challenges of this era is processing massive amounts of data and extracting biological meaning. Effective integration of these datasets constitutes another challenge when trying to gain insights into cellular, molecular, and biological systems under study [1]. After the primary bioinformatics analysis of omics data, the biomedical researcher is given a list of statistically significant molecules (e.g., RNAs, proteins, metabolites). Usually, these lists can contain hundreds or thousands of “interesting” or differentially regulated molecules. Thus, an essential part of omics data analysis and interpretation includes pathway and/or network analyses, which provide context for explaining the research findings or support for the hypothesis generation or narrowing process. Consequently, almost all omics studies include a pathway or network analysis of the data.

**Figure 1:**
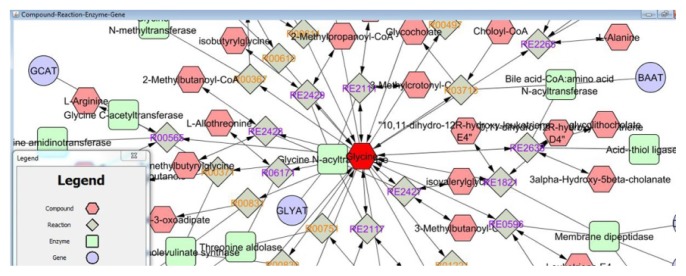
Example Cytoscape network

Cytoscape software is one of the most popular tools for network analysis and visualization [2]. Cytoscape helps to visually integrate the network with expression profiles, phenotypes, and other molecular states and to link the network to databases of functional annotations [3]. For example, suppose a researcher desires more information about the compound glycine, including its related genes and compounds. Using the MetScape app for Cytoscape [4]; the researcher builds a network of glycine and its related compounds, genes, reactions, and enzymes. From this network, the researcher begins to understand glycine’s relationship to other compounds (e.g., L-alanine) and genes (e.g., GLYAT and BAAT) (Figure 1). Additionally, the MetScape app provides a list of pathways included in the network, such as the bile acid biosynthesis pathway, thus providing valuable information about the relationship between molecular entities. The researcher can create a subnetwork of a chosen pathway for further analysis and understanding. If using experimental data, the researcher can compare networks across different treatments or periods of time to visualize and understand molecular changes.

Librarians determine the information and data needs of their users and establish ways to meet those needs. Although exact needs can vary between institutions and organizations, definite patterns can be discerned, such as the demand for pathway and network analysis tools. Discussion across institutions can lead to a better understanding of these patterns and the appropriate services to provide. Cytoscape is one of many tools for network analysis and visualization, each having their own strengths and weaknesses. However, researchers can find the process of learning and using new tools like Cytoscape to be time consuming and frustrating. As a result, it is often advantageous for them to seek help from a knowledgeable source, which is where librarians can play a valuable role by offering training, documentation, and consultation. However, with the constantly increasing development of bioinformatics analysis tools, it is challenging for one person to be an expert in every tool. One method of overcoming this challenge is to collaborate with peers at other institutions, which can enable librarians to take advantage of existing expertise, learn from each other, and ultimately provide a wider range of services.

## STUDY PURPOSE

The objective of this case study is to describe a librarian-led, interinstitutional collaborative effort to provide training in Cytoscape network analysis and visualization software to biomedical researchers. The authors discuss the value of this collaboration as a means for filling end-user training and other knowledge gaps in order to keep up with the rapidly changing field of bioinformatics.

## CASE PRESENTATION

### Description of Cytoscape

Cytoscape is a free, open-source software tool with an active international community [3]. It is platform-independent, meaning that it can be used on a Mac, PC, or Linux machine. This software has a plugin architecture that allows anyone to create an extension, referred to as an “app,” to add functionality to the core. This type of architecture is advantageous as it greatly expands the capabilities of the software. For example, there are apps that allow importing data directly from other databases, such as the Reactome pathway database [5], while other apps run clustering algorithms on existing data. There are also literature mining apps and functional enrichment apps [6]. Cytoscape allows the user to change the color, shape, and size of the network components to highlight certain information within the network, as well as to create subnetworks and merge networks. As a result, in addition to being used for data analysis and discovery, Cytoscape is often used to visualize data to make the data more understandable for use in a publication. The flexibility, diverse functionality, plugin architecture, and free nature of this tool add to its popularity.

### Initial webinar

The Yale University Harvey Cushing/John Hay Whitney Medical Library (CWML) biomedical sciences research support librarian attended the “Network Visualization Tools” continuing education course at MLA ‘13, the 2013 Medical Library Association (MLA) annual meeting, which was co-taught by the University of Michigan (U-M) bioinformationist. This course focused on two open-source network visualization tools: Cytoscape and visANT. Two years later, the Yale librarian and U-M bioinformationist reconnected through an alumni group of librarians who attended the “Librarians’ Guide to NCBI” workshop at the National Institutes of Health. This group holds monthly remote meetings to learn about new activities at the National Center for Biotechnology Information (NCBI) and to share bioinformatics-related work being done by librarians at different institutions. The U-M bioinformationist gave a brief presentation on Cytoscape during one of these group meetings, which prompted the Yale librarian to consider his researchers’ needs for instruction on visualization tools like Cytoscape.

To determine Yale biomedical researchers’ level of interest in Cytoscape training, the U-M bioinformationist offered a 1-hour webinar (“Introduction to Cytoscape: Network Visualization Software”) to introduce Yale researchers to the software. Invitations to the webinar were sent to all departments using an internal Yale School of Medicine messaging system. The sessions were advertised on CWML web pages, including the blog, calendar of classes, and a banner on the home page, as well as in the Yale School of Medicine calendar of events. In addition, an email was sent to a list of Yale affiliates (over 1,000 members) who are interested in end-user bioinformatics resources and tools. The webinar included a live demonstration with screenshots to cover a wider span of functionality in a limited amount of time. This webinar was presented via Adobe Connect. Registration for the session reached maximum capacity with 22 attendees and an additional 19 individuals waitlisted.

The success of the Cytoscape webinar made it clear that there was both interest in and a need for instruction on visualization software at Yale University. However, Cytoscape has a steep learning curve due to its complexity, and skill acquisition is greatly enhanced by hands-on instruction. Therefore, given the U-M bioinformationist’s instructional expertise in Cytoscape [7], it made sense for the Yale librarian to collaborate with the U-M bioinformationist on additional Cytoscape instruction opportunities, freeing him to focus on locally supported bioinformatics resources and services.

### Attainment of funding

Although librarians are aware of their communities’ needs for specific training, they often do not have the funding available to bring in appropriate experts to provide this instruction. Because the CWML did not have the funds to bring the U-M bioinformationist to the campus, an application was submitted for the Jay Daly Technology Grant (approximately $1,000 USD), offered by the North Atlantic Health Sciences Libraries, a regional chapter of MLA, that represents Connecticut in addition to other North Atlantic states. This grant allows recipients to acquire, implement, and become knowledgeable about new technologies; to improve services or access in their institutions; and to draw attention to the librarians’ value to their institutions by providing them with tools that allow them to evolve in their professional role, among others. The authors drafted a joint proposal, submitted by the Yale librarian, to bring the U-M bioinformationist to Yale University to provide onsite Cytoscape training. In October 2015, the grant was awarded to the Yale librarian and provided the funds to cover the U-M bioinformationist’s travel expenses for offering a 1-day workshop at Yale University.

### Onsite hands-on instruction

Once the need was determined and funding was secured, the Yale librarian and U-M bioinformationist collaborated to schedule a 1-day Cytoscape workshop at Yale University. This workshop consisted of 2 sessions, each covering different Cytoscape features and functionalities. “Cytoscape Part 1: Going from Raw Data to a Publishable Image” (150 minutes) covered general features of the Cytoscape core software, and “Cytoscape Part 2: Cytoscape Apps with a Focus on MetScape” (180 minutes) covered several apps and focused on metabolomics, which is the study of small compounds involved in metabolism. Although both sessions started with background slides, the lecture consisted of less than 25% of the workshop. Rather, the majority of each session consisted of hands-on training where attendees followed along with the instructor. A few brief exercises were also mixed in for attendees to work on independently. The sessions were developed in a way that made them independent from each other, allowing researchers to attend either session or both sessions, depending on their research, interests, and proficiency level with the software. Both sessions were free and open to Yale School of Medicine biomedical researchers and medical librarians in the New England Region.

Eighteen individuals attended the first session, and 6 attended the second session. Given the more specific nature of the second session, the decline in attendance was expected. Combining the webinar and hands-on sessions, postdoctoral fellows were the most common type of attendee (42.7%), followed by associate research scientists (21.3%), graduate students (16.0%), faculty (9.3%), and staff (6.7%). The top represented departments were internal medicine (13.3%), immunobiology (9.3%), cell biology (6.6%), pathology (6.6%), and genetics (5.3%). However, individuals affiliated with 30 additional departments attended the sessions.

To make the training sessions more relevant to attendees, the Yale librarian and U-M bioinformationist collaborated on tailoring the content of the instruction sessions based on the local bioinformatics resources and tools that the CWML offered. For example, the U-M bioinformationist usually provides instruction on several pathway and gene ontology enrichment apps, such as BiNGO [8] and ReactomeFIPlugIn [9], that work with the Cytoscape software. However, since the CWML provides site licenses to several other commercial bioinformatics software for enrichment and pathway analysis, such as Ingenuity Pathway Analysis, the U-M bioinformationist redesigned the training sessions to teach other equally valuable Cytoscape apps and features, such as MCode, a clustering app [10], and SocialNetwork, an app used to create networks of publication coauthors [11]. This put the focus of the sessions on functionality and analyses that differed from those already available to Yale researchers through subscription resources.

The Yale librarian took care of the onsite logistics, such as room reservation, promotion, and attendee registration. Promotion for the onsite trainings followed the same process as the webinar. The Yale librarian worked with the U-M bioinformationist to ensure that registrants were provided all necessary information (e.g., computer software requirements, presentation materials, working files) in advance of the session. The U-M bioinformationist created step-by-step handouts that were provided to attendees, allowing them to follow along more easily. Attendees could also refer back to the handouts when exploring Cytoscape after completing the sessions.

To obtain feedback, an online evaluation form was provided to all attendees after the webinar and hands-on sessions. Five attendees filled out the evaluation form after the hands-on sessions, and 1 attendee filled out the evaluation form after the webinar. Of these 6 respondents, 3 were postdoctoral fellows, 1 was a faculty member, 1 was a staff member, and 1 was a graduate student. The respondents rated the quality of the instructor as excellent; the instructor’s knowledge between excellent (4 attendees) and good (2 attendees); the instructor’s responsiveness to questions between excellent (5 attendees) and good (1 attendee); and their knowledge of the material after the workshop between excellent (3 attendees), good (2 attendees), and fair (1 attendee). In response to the question “What did you like most about the workshop?”, 2 respondents noted the interactiveness of the hands-on sessions: “interactive session with hands-on training” and “the interactiveness was great; we could work along while the instructor was going.” This reinforced the value and need for hands-on instruction for visualization software such as Cytoscape.

## DISCUSSION

Although some libraries have been offering bioinformatics services for over a decade [12–14], many institutions are still working to build up their bioinformatics programs. Many librarians provide instruction on resources from NCBI, the European Bioinformatics Institute resources, and others, but the interdisciplinary nature of bioinformatics adds complexity that can make library services challenging. In particular, a survey at the University of Southern California showed that researchers highly value bioinformatics workshop training but noted a gap in training for appropriate analysis tools [15].

Here, the authors present Cytoscape as an example of interinstitutional collaboration to provide end-user bioinformatics training between the Yale CWML and the U-M Taubman Health Sciences Library. One challenge of bioinformatics instruction is keeping up with the rapidly changing field when one is not a bioinformatics researcher, and thus more training opportunities are needed for those who teach bioinformatics [16]. The Yale librarian attended the Cytoscape webinar and hands-on sessions to receive the necessary training to provide additional reference, consultations, and training on Cytoscape at the Yale School of Medicine. The Yale librarian’s increased knowledge of Cytoscape allowed continued collaboration with the U-M bioinformationist, as they could work together to answer more challenging questions and share instruction and learning objects. For example, the two librarians recently used remote video technology to jointly conduct a consultation with a Yale biomedical researcher using Cytoscape for metabolomics data analysis. Despite the geographical separation between Yale University and U-M, video technology (Zoom) allowed the two librarians to share computer screens and synchronously work with the researcher, making this interinstitutional consultation efficient and productive.

This collaboration was beneficial for both librarians, their libraries, and their institutions by increasing the wealth of knowledge about tools and strengthening the services provided at both institutions. Some logistical lessons were also learned from this experience. For example, distributing the evaluation form before attendees left the room might have led to a greater response rate. In addition, had the webinar and onsite sessions been recorded, they would be available to those who were unable to attend the live sessions, further increasing the reach of these instruction sessions.

Adding value to the onsite visit to Yale, the U-M bioinformationist gave a presentation on her institution, library, and work responsibilities to the librarians at the CWML. The U-M bioinformationist also had the opportunity to tour the CWML, network with other librarians, and learn about their services and roles. These types of interactions are important for learning about different types of expertise and service models to foster further collaboration among libraries. This collaboration furthered the U-M bioinformationist’s role as an expert in Cytoscape instruction, while also putting the CWML in a leadership position in terms of providing support for analyzing and visualizing omics data. This collaboration was a successful means for librarians to fill end-user training gaps in rapidly changing fields such as bioinformatics, and the positive outcomes suggest that other librarians should seek out similar relationships. Creating a librarian network of collaborators would significantly strengthen librarian-provided bioinformatics services, skills, and training.

## References

[1] Joyce AR, Palsson BØ (2006). The model organism as a system: integrating ‘omics’ data sets. Nat Rev Mol Cell Bio.

[2] Villaveces JM, Koti P, Habermann BH (2015). Tools for visualization and analysis of molecular networks, pathways, and omics data. advances and applications in bioinformatics and chemistry. Adv Appl Bioinform Chem.

[3] Shannon P, Markiel A, Ozier O, Baliga NS, Wang JT, Ramage D, Amin N, Schwikowski B, Ideker T (2003). Cytoscape: a software environment for integrated models of biomolecular interaction networks. Genome Res.

[4] Karnovsky A, Weymouth T, Hull T, Tarcea VG, Scardoni G, Laudanna C, Sartor MA, Stringer KA, Jagadish HV, Burant C, Athey B, Omenn GS (2012). Metscape 2 bioinformatics tool for the analysis and visualization of metabolomics and gene expression data. Bioinformatics.

[5] Fabregat A, Sidiropoulos K, Garapati P, Gillespie M, Hausmann K, Haw R, Jassal B, Jupe S, Korninger F, McKay S, Matthews L, May B, Milacic M, Rothfels K, Shamovsky V, Webber M, Weiser J, Williams M, Wu G, Stein L, Hermjakob H, D’Eustachio P (2016). The Reactome pathway knowledgebase. Nucleic Acids Res.

[6] Saito R, Smoot ME, Ono K, Ruscheinski J, Wang PL, Lotia S, Pico AR, Bader GD, Ideker T (2012). A travel guide to Cytoscape plugins. Nat Methods.

[7] Brandenburg MD, Song J (2012). Broadening instructional scope with network visualization. J Med Libr Assoc.

[8] Maere S, Heymans K, Kuiper M (2005). BiNGO: a Cytoscape plugin to assess overrepresentation of gene ontology categories in biological networks. Bioinformatics.

[9] Wu G, Feng X, Stein L (2010). A human functional protein interaction network and its application to cancer data analysis. Genome Biol.

[10] Bader GD, Hogue CW (2003). An automated method for finding molecular complexes in large protein interaction networks. BMC Bioinformatics.

[11] Kofia V, Isserlin R, Buchan AM, Bader GD (2015). Social Network: a Cytoscape app for visualizing co-authorship networks. F1000Res.

[12] Lyon JA, Tennant MR, Messner KR, Osterbur DL (2006). Carving a niche: establishing bioinformatics collaborations. J Med Libr Assoc.

[13] Wang L, Lipsey K, Murray C, Prendergast N, Schoening P (2007). The Bioinformatics Program at Washington University’s Bernard Becker Medical Library: making it happen. Med Ref Serv Q.

[14] Tennant MR (2005). Bioinformatics librarian: meeting the information needs of genetics and bioinformatics researchers. Ref Serv Rev.

[15] Li M, Chen YB, Clintworth WA (2013). Expanding roles in a library-based bioinformatics service program: a case study. J Med Libr Assoc.

[16] Cummings MP, Temple GG (2010). Broader incorporation of bioinformatics in education: opportunities and challenges. Briefings Bioinform.

